# SARS‐CoV‐2 convalescent plasma for the controlled clinical trial “COVIC‐19”: Experience from collection of very high‐titer plasma from superimmunized individuals

**DOI:** 10.1111/trf.18394

**Published:** 2025-09-12

**Authors:** Simone Hoffmann, Alina Seidel, Carolin Ludwig, Christiane Vieweg, Rebecca Müller, Henrike Hofmann, Bernd Jahrsdörfer, Patrick Wuchter, Harald Klüter, Michael Schmidt, Matthias Johnsen, Thomas Burkhardt, Thomas Appl, Eva Schrezenmeier, Jan Münch, Pierre Tiberghien, Hubert Schrezenmeier, Sixten Körper

**Affiliations:** ^1^ Institute of Clinical Transfusion Medicine and Immunogenetics Ulm, German Red Cross Blood Transfusion Service Baden‐Württemberg‐Hessen and University Hospital Ulm; and Institute of Transfusion Medicine, University Ulm Ulm Germany; ^2^ Institute of Molecular Virology Ulm University Medical Center Ulm Germany; ^3^ Institute of Transfusion Medicine and Immunology Medical Faculty Mannheim, Heidelberg University, German Red Cross Blood Transfusion Service Baden‐Württemberg – Hessen Mannheim Germany; ^4^ Institute of Transfusion Medicine and Immunohematology German Red Cross Blood Transfusion Service Baden‐Württemberg – Hessen Frankfurt Germany; ^5^ Institute of Transfusion Medicine Dresden German Red Cross Blood Transfusion Service Nord‐Ost gGmbH Dresden Germany; ^6^ Institute of Transfusion Medicine Plauen German Red Cross Blood Transfusion Service Nord‐Ost gGmbH Plauen Germany; ^7^ Department of Nephrology and Medical Intensive Care Charité‐Universitätsmedizin Berlin Berlin Germany; ^8^ UMR 1098 Right, Inserm, Université Marie et Louis Pasteur Besançon France; ^9^ Etablissement Francais du Sang La Plaine Saint‐Denis France

**Keywords:** blood component preparations, donors, FFP transfusion

## Abstract

**Background:**

COVID‐19 convalescent plasma (CCP) is a treatment option for COVID‐19. Understanding of donor and product characteristics is important for optimization of CCP therapy. We report the experience of collection of very high‐titer CCP for the trial “A randomized open‐label trial of early, very high‐titer convalescent plasma therapy in clinically vulnerable individuals with mild COVID‐19” (COVIC‐19) (NCT05271929).

**Study Design and Methods:**

Individuals who had recovered from COVID‐19 and had ≥1 dose of a SARS‐CoV‐2 vaccine were recruited as donors for CCP. Anti‐SARS‐CoV‐2 antibodies were measured by ELISA, and neutralization capacity against SARS‐CoV‐2 variants was assessed in surrogate and pseudovirus‐neutralization assays. Correlation of antibody titers with donor characteristics and antibody kinetics was analyzed.

**Results:**

We recruited 688 potential donors. 41.4% of individuals had antibody concentrations of ≥4000 BAU/ml (anti‐SARS‐CoV‐2 IgG ELISA [QuantiVac]). Concentrations did not significantly differ by sex or ABO type, but were higher among those who had received at least three vaccinations. Highest titers were observed in those with a breakthrough infection after two vaccinations, followed by a booster (median 5374 BAU/mL) or breakthrough infection after the 3rd or 4th vaccination (median 3846 BAU/mL). Ultimately, 172 eligible individuals donated CCP with a median concentration of 6858 BAU/mL (range 4015–22,923 BAU/mL).

**Discussion:**

We demonstrate the feasibility of the collection of very high‐titer CCP products under a harmonized protocol for a randomized clinical trial, but it requires substantial donor selection, appropriate antibody assays, rapid succession of screening, and apheresis to take advantage of the short period of very high antibody concentrations.

**Abbreviations**
ATCC: American Type Culture Collection
BAU: binding antibody units
CCP: COVID‐19 convalescent plasma
COVIC‐19: a randomized open‐label trial of early, very high‐titer convalescent plasma therapy in clinically vulnerable individuals with mild COVID‐19
COVID‐19: Coronavirus Disease 2019
ELISA: enzyme‐linked immunosorbent assay
FDA: Food and Drug Administration
FLuc: firefly luciferase
Geo.mean: geometric mean
h: hour
HLA: human leukocyte antigen
HNA: human neutrophile antigen
IMP: Investigational Medicinal Product
IQR: interquartile range
mRNA: messenger RNA
NT50: serum dilution on cell resulting in 50% pseudovirus neutralization
PBS: phosphate‐buffered saline
PCR: polymerase chain reaction
RNA: ribonucleic acid
SARS‐CoV‐2: severe acute respiratory syndrome coronavirus type 2
SoC: standard‐of‐care
VOC: variant of concern
VSV: vesicular stomatitis virus
VSV‐G: VSV native glycoprotein

## 1 INTRODUCTION

COVID‐19 convalescent plasma (CCP) was considered very early in the SARS‐CoV‐2 pandemic as one of the treatment options for severe COVID‐19.^1^ However, clinical data on efficacy have been heterogenous to date. It became evident that CCP is not beneficial if anti‐SARS‐CoV‐2 antibody concentrations are low, if CCP is administered late or in advanced disease stages, and to patients who have already mounted an antibody response against SARS‐CoV‐2 at the time of CCP transfusion.^2^, ^3^

The COVIC‐19 study^4^, ^5^, ^6^ adopted a novel treatment approach drawing on the lessons learned from previous studies: (i) CCP with very high levels of SARS‐CoV‐2 antibodies (>4000 BAU/ml measured in the QuantiVac [Euroimmun] or >20,000 IU/mL measured in the anti‐SARS‐CoV‐2 IgG antibody CLIA [Roche]) from superimmunized donors (i.e., previous SARS‐CoV‐2 infection and vaccination against SARS‐CoV‐2), (ii) outpatient CCP treatment early after the onset of symptoms (within 5 days), (iii) treatment of vulnerable persons, namely, immunocompromised patients, and (iv) analysis of immune escape (virus evolution in patients).

The trial enrolled 120 immunocompromised outpatients with mild COVID‐19 within 7 days from symptom onset. Patients were randomized 1:1 to receive high‐titer CCP in addition to standard‐of‐care (SoC) or SoC alone in an open‐label design. The primary endpoint was hospitalization for COVID‐19 progression or death by Day 28. None of the CCP‐treated patients reached the primary endpoint, compared with 8.6% in the SoC group (*p* = 0.027, Fisher's exact test). CCP was well tolerated, led to passive antibody transfer including neutralizing effects against emerging variants but did not affect viral evolution within patients.^5^

The exclusive use of CCP units which exceed the predefined thresholds is a key feature of this trial. The titers required for the trial were substantially higher than those typically achieved by the vast majority of convalescent individuals without further immunization by vaccination and, vice versa, individuals who have been immunized by vaccination alone do not reach. Only a proportion of (repeatedly) vaccinated and convalescent individuals reach these thresholds.^7^, ^8^, ^9^, ^10^ Therefore, the COVIC‐19 trial only included superimmunized donors with infection *and* vaccination which mount a higher antibody concentration and an enhanced neutralizing breadth.^11^, ^12^, ^13^ Here, we sought to address the following questions:

- Is it feasible to obtain sufficient high‐titer CCP units for a clinical trial?
- Is a very high anti‐SARS‐CoV‐2 antibody level of these donors associated with other characteristics, which might help to optimize selection of superimmunized donors?
- Do superimmunized donors sustain high anti‐SARS‐CoV‐2 levels to enable their repeated recruitments?

## 2 STUDY DESIGN AND METHODS

### 2.1 Donors

CCP donors were recruited within the clinical trial “COVIC‐19” (EUdraCT Nummer: 2021–006621‐22; clinicaltrials.gov: NCT05271929).^4^, ^5^ The trial was approved by the Ethics Committee of Ulm University (Number 41/22; 14 February 2022) and by the Paul‐Ehrlich‐Institute (Federal Institute for Vaccines and Biomedicines, Langen, Germany) on 8 February 2022. Plasma donations and blood samples were collected after informed consent.

The detailed criteria for acceptance to be included as a CCP donor were as follows:

- Infection with SARS‐CoV‐2, documented by PCR.
- Minimum of 2 weeks since resolution of symptoms of the SARS‐CoV‐2 infection.
- Documentation of at least one dose of SARS‐CoV‐2 vaccination with a minimum interval between the first vaccination and the first plasmapheresis of 3 weeks.
- Negative test for antibodies against HLA class I, class II, and HNA‐antigens irrespective of sex and previous pregnancies.
- Anti‐SARS‐CoV‐2 antibody concentration above 4000 BAU/mL in the QuantiVac or above 20,000 IU/mL in the Elecsys assay. A donor should continue plasma donation only in case of antibody titers above cut‐off.

There were no restrictions on the order of immunization events (infection or vaccination) as long as the antibody thresholds were met. In 2021, a person in Germany was considered fully vaccinated against COVID‐19 after receiving two doses of any approved vaccine. As of October 2022, a person was only considered fully vaccinated if they had received three vaccine doses or two doses plus proof of a past SARS‐CoV‐2 infection confirmed by a PCR test. However, it was not a requirement for donors to have completed their basic immunization. In addition to these study‐specific eligibility criteria, each donation had to meet the standard criteria for approval as a plasma donor according to the national German Guidelines (“Richtlinie Hämotherapie”^14^) as summarized in a previous publication on the plasma donation for the randomized clinical trial CAPSID.^15^ The procedure including frequency and intervals was performed accordingly.^14^, ^15^

Between November 2021 and March 2023, potential donors were recruited by social media calls and invited for a screening visit, where the qualification as a donor was determined by medical history, physical examination, and laboratory analyses. Donations from eligible donors took place between January 2022 and May 2023.

### 2.2 Quantification of anti‐SARS‐CoV‐2 antibodies and neutralization assays

Serum samples of CCP donors were analyzed by two commercially available assays according to the instructions of the manufacturer (anti‐SARS‐CoV‐2‐QuantiVac‐ELISA (IgG), Euroimmun and Elecsys Anti‐SARS‐CoV‐2 S, Roche). To ensure the quality of the IMP in terms of a very high antibody concentration, the SARS‐CoV‐2 antibody concentration was determined again at each plasmapheresis.

Production of rhabdoviral pseudotypes has been previously described.^5^, ^16^ In brief, 293 T cells (ATCC no. CRL‐3216) were transfected with expression plasmids encoding SARS‐CoV‐2 spike variants B.1,^17^ BA.1,^18^ BA.2,^19^ or BA.5,^20^ BQ.1.1,^21^ XBB.1.5,^22^ BA.2.86, and EG.5.1^23^ (kindly provided by Stefan Pöhlmann, Infection Biology Unit, German Primate Center, Göttingen, Germany) by TransIT LT‐1 (Mirus). One day after transfection, cells were inoculated with a replication‐deficient vesicular stomatitis virus (VSV) vector in which the genetic information for its native glycoprotein (VSV‐G) was replaced by genes encoding enhanced green fluorescent protein and firefly luciferase (FLuc), kindly provided by Gert Zimmer, Institute of Virology and Immunology, Mittelhäusern, Switzerland,^24^ and incubated for 2 h at 37°C. The inoculum was removed, cells were washed with phosphate‐buffered saline (PBS) and fresh medium containing anti‐VSV‐G antibody (I1‐hybridoma cells; ATCC no. CRL‐2700) was added to block remaining VSV‐G carrying particles. After16–18 h, supernatants were collected and centrifuged (2000 x g, 10 min, room temperature) to clear cellular debris. Samples were aliquoted and stored at −80°C.

The pseudovirus neutralization experiments were performed as previously described [83]. In brief, Vero E6 cells were seeded in 96‐well plates 1 day prior (6000 cells/well, 2.5% FCS). Sera were heat‐inactivated (56°C, 30 min) and serially titrated (4‐fold titration series with seven steps + buffer‐only control) in PBS, with undiluted pseudovirus stocks added (1:1, v/v) and the mixtures incubated for 30 min at 37°C before being added to cells in duplicates (final on‐cell dilution of sera: 20, 80, 320, 1280, 5120, 20480, 81920‐fold). After an incubation period of 16–18 h, transduction efficiency was analyzed. For this, the supernatant was removed, and cells were lysed by incubation with Cell Culture Lysis Reagent (Promega) at room temperature. Lysates were then transferred into white 96‐well plates, and luciferase activity was measured using a commercially available substrate (Luciferase Assay System, Promega) and a plate luminometer (Orion II Microplate Luminometer, Berthold). For the analysis of raw values (relative luminescence units per s, RLU/s), the background signal of untreated cells was subtracted, and values were normalized to cells inoculated with pseudovirus preincubated with PBS only. Results are given as serum dilution on cell resulting in 50% pseudovirus neutralization (NT50), calculated by nonlinear regression ([Inhibitor] vs. normalized response—variable slope) in GraphPad Prism Version 9.1.1.

Neutralization capacity of transfused CCP was also assessed using GenScript surrogate neutralization test against wildtype and Omicron SARS‐CoV‐2 (GenScript Biotech, cat. no L00847‐C, L00847‐C + Z03730‐2).

### 2.3 Statistical analyses

Summarized data are expressed as medians and interquartile ranges (IQR). Statistical differences were assessed using the Mann–Whitney test or the Kruskal‐Wallis test with correction for multiple comparisons by Dunn's test. *p*‐values <0.05 were considered to indicate statistical significance.

## 3 RESULTS

### 3.1 Donor recruitment

**FIGURE 1:**
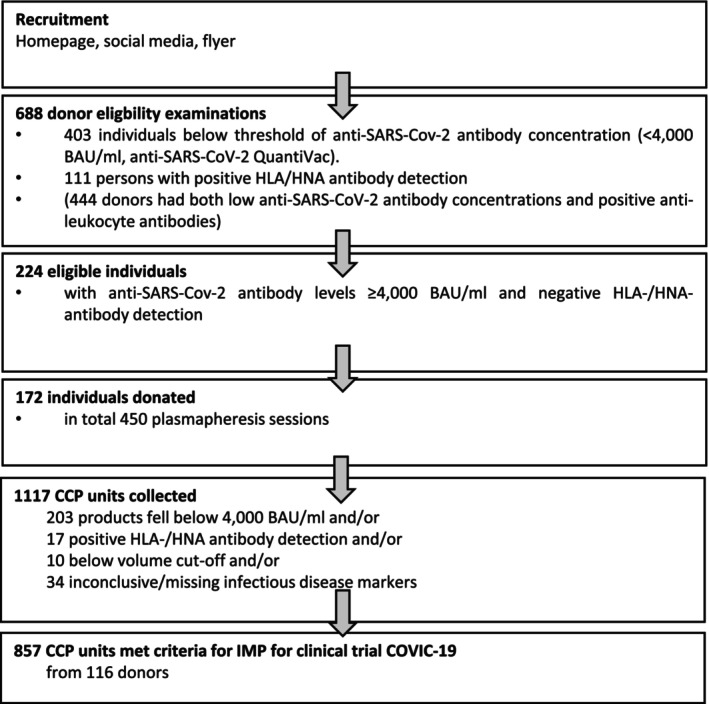
Overview of the donor recruitment process up to the approved investigational medicinal product for use in the clinical trial COVIC‐19.

A total of 688 potential donors were recruited between November 2021 and March 2023. An overview of the donor recruitment process up to the release of the investigational medicinal product (IMP) is shown in Figure 1.

The vast majority of individuals willing to donate (97.1%) had one documented SARS‐CoV‐2 infection and had at least three vaccinations (72.2%). Furthermore, the interval between eligibility check and last immunization event was brief, with a median of 54 days. Mainly RNA vaccines have been used (Table 1). The date of infection of the donors is known, and we assumed that the variant most prevalent in the general population according to surveillance data at that time was most likely to have caused the infection. According to the date of infection, most individuals were infected during the B.1.617.2 (14.1%), BA.1 (25.7%), and BA.2 (31.8%) waves. The variant has not been confirmed by molecular virus testing.

**TABLE 1:** Demographics.

Characteristic	Willing to donate	Eligible to donate	Donors^a^
	*n* = 688	*n* = 224	*n* = 172
Sex (*n*, %)			
Male	282 (41%)	95 (42.4%)	77 (44.8%)
Female	406 (59%)	129 (57.6%)	95 (55.2%)
Age (years)			
Median	34.4	34.7	35.3
IQR	21.6	23.5	24.3
AB0 (*n*, %)			
A	256 (37.2)	81 (36.2%)	52 (32.2%)
B	113 (16.4%)	40 (17.9%)	30 (17.4%)
AB	97 (14.1%)	25 (11.2%)	24 (14%)
0	216 (31.4%)	76 (33.9%)	66 (38.4%)
Number of infections (*n*, %)			
1	670 (97.4%)	222 (99.1%)	165 (95.9%)
2	16 (2.3%)	2 (0.9%)	7 (4.1%)
3	2 (0.3%)	0 (0%)	0 (0%)
Vaccination doses (*n*, %)			
1	28 (4.1%)	4 (1.8%)	4 (2.3%)
2	163 (23.7%)	34 (15.2%)	24 (14.0%)
3	489 (71.1%)	182 (81.3%)	139 (80.8%)
4	7 (1.0%)	4 (1.8%)	5 (2.9%)
5	1 (0.1%)	0 (0%)	0 (0%)
SARS‐CoV‐2 variant (*n*, %)			
B.1	86 (12.5%)	20 (8.9%)	17 (9.9%
B.1.1.7	40 (5.8%)	4 (1.8%)	7 (4.1%)
B.1.617.2	97 (14.1%)	37 (16.5%)	30 (17.4%)
BA.1	177 (25.7%)	65 (29.0%)	44 (25.96%)
BA.2	219 (31.8%)	74 (33.0%)	54 (31.4%)
BA.5	66 (9.6%)	23 (10.3%)	18 (10.5%)
N/A	3 (0.4%)	1 (0.4%)	2 (1.2%)
HLA/HNA‐antibodies (*n*, %)			
Positive (all)	111 (16.1%)	0 (0%)	5 (2.9%)
Male	35 (5.1%)	0 (0%)	4 (2.3%)
Female	76 (11.0%)	0 (0%)	1 (0.6%)
Negative	505 (73.4%)	224 (100%)	166 (96.5%)
n.a.	72 (10.5%)	0 (0%)	1 (0.6%)
≥4.000 BAU/mL (*n*, %)			
Yes	285 (41.4%)	224 (100%)	126 (73.3%)
No	403 (58.6%)	0 (%)	46 (26.7%)
Interval since last infection (days)			
Median	63.0	57.0	85.0
IQR	82.5	61.25	74.5
Interval since last vaccination (days)			
Median	138	135	168.7
IQR	111.7	115.5	118.0
Interval since last immunization event (days)			
Median	54.0	50.0	70.0
IQR	46.0	37.0	49.0
Vaccination (homologous regime) (*n*, %)	568 (82.6%)	190 (84.8%)	141 (82.0%)
mRNA	557 (98.1%)	187 (98.4%)	139 (98.6%)
Vector	19 (1.8%)	2 (1.1%)	1 (0.7%)
Protein	1 (0.2%)	1 (0.5%)	1 (0.7%)
Vaccination (heterologous regime) (*n*, %)	120 (17.4%)	34 (15.2%)	31 (18.0%)
Vector/mRNA	17 (14.2%)	7 (20.6%)	5 (16.1%)
Vector/mRNA/mRNA	80 (66.7%)	21 (61.8%)	19 (61.3%)
Vector/mRNA/mRNA/mRNA	2 (1.7%)	1 (2.9%)	1 (2.9%)
Vector/mRNA/mRNA/mRNA/mRNA	1 (0.8%)	1 (2.9%)	1 (2.9%)
Vector/Vector/mRNA	19 (15.8%)	4 (11.8%)	4 (12.9%)
mRNA/mRNA/Vector	1 (0.8%)	0 (0%)	0 (0%)

^a^ Results at time of donation, i.e., after negative test during screening before some donors had positive results at time of donation.

Further demographic characteristics are summarized in Table 1.

### 3.2 Anti‐SARS‐CoV‐2 antibody concentrations at eligibility check

The median SARS‐CoV‐2 antibody titer of those willing to donate was 3476 BAU/mL (IQR: 2193–5645 BAU/mL) as measured in the QuantiVac ELISA. Only 41.4% of those willing to donate had an anti‐SARS‐CoV‐2 antibody concentration above the threshold of 4000 BAU/mL. In addition, donors needed a negative test result for anti‐HLA and anti‐HNA antibodies. Seventy‐three percent of the candidates did not carry anti‐HLA and anti‐HNA antibodies. Including the negative test result for anti‐HLA and anti‐HNA antibodies and the required anti‐SARS‐CoV‐2 antibody titer, only 32.5% of the screened donors fulfilled the specified criteria.

**FIGURE 2:**
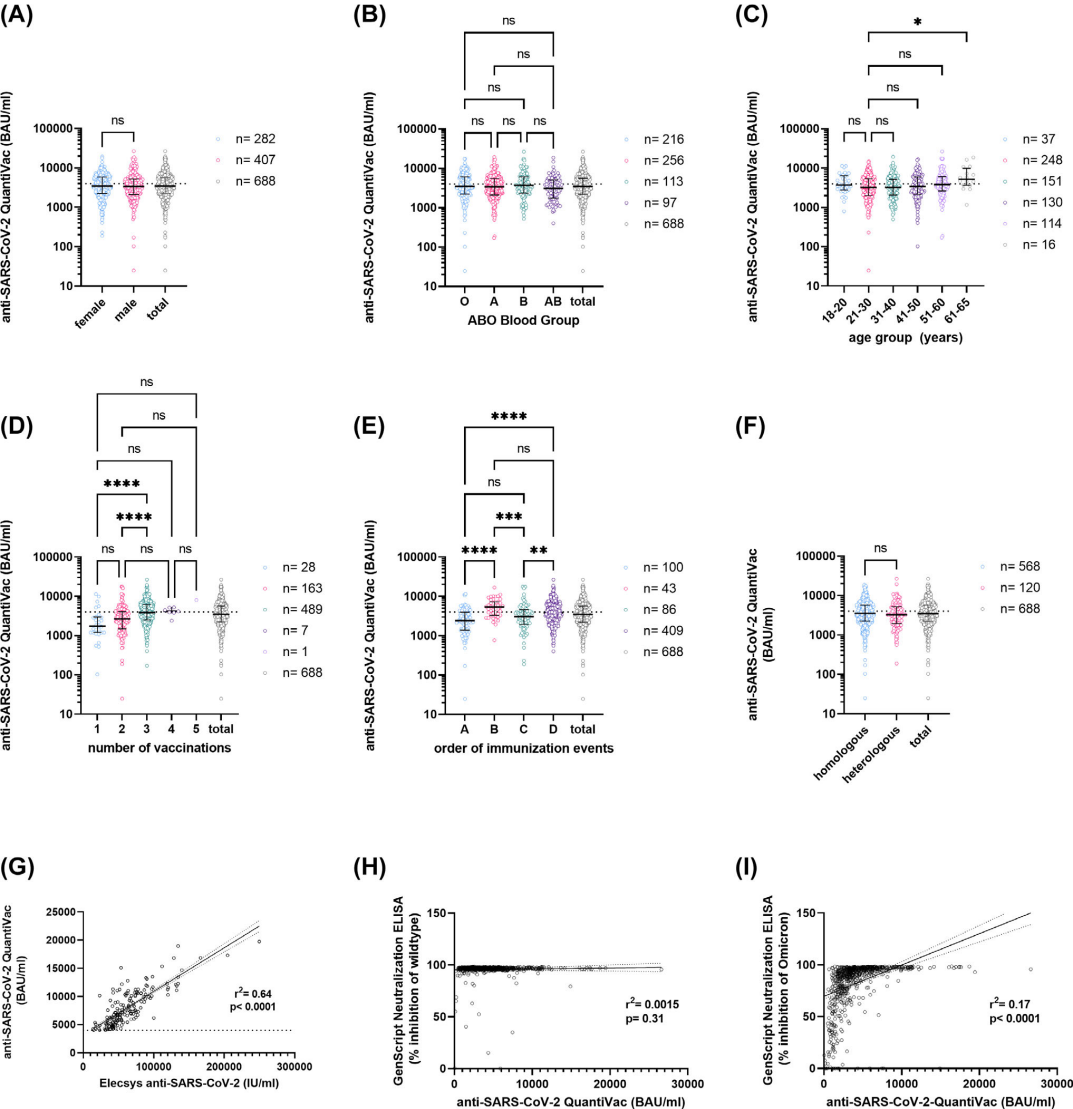
Characterization of prospective donors during eligibility check. anti‐SARS‐CoV‐2 antibody concentrations measured by anti‐SARS‐CoV‐2‐QuantiVac‐ELISA (IgG) stratified by sex (A), AB0 blood type (B), age (C), number of vaccinations (D), sequence of immunization events (A = infection before first vaccination, B = infection between second and booster, C = infection after two vaccinations, D = infection after booster vaccination) (E), and vaccination scheme (F). Dotted horizontal line indicates threshold of 4000 BAU/ml. The horizontal solid lines indicate the median and the error bars the interquartile ranges. Statistical differences were assessed using Mann–Whitney (A) or Kruskal‐Wallis test followed by Dunn's test for correction of multiple comparisons (B‐F). *p*‐values <0.05 were considered statistically significant; ns = not significant, *p < 0.05; **p < 0.05; ****p < 0.0005). For (C) we have chosen the age group 21–30 as reference group. Correlations between the serological assays anti‐SARS‐CoV‐2 QuantiVac and Elecsys (G) and between anti‐SARS‐CoV‐2 QuantiVac and the GenScript Neutralization assays against wildtype (H) and Omicron (I) were assessed. The solid lines show the linear regression and the dotted lines the 95% confidence interval. [Color figure can be viewed at wileyonlinelibrary.com]

Sex, age, and blood group had no influence on the antibody concentrations (Figure 2A–C); however, the antibody titer increased with the number of vaccinations (Figure 2D). The titers after one or two vaccinations do not differ significantly. However, the concentration after three vaccinations compared with only one, or three vaccinations compared with two vaccinations, is significantly higher in each case (Figure 2D). The sequence of immunization events had an influence on the anti‐SARS‐CoV‐2 antibody concentrations (Figure 2D). Based on the various combinations of vaccination and infection order and frequency, distinct sequence groups were established. The analysis was limited to the sequence groups that were present in at least 5% of the prospective donors. Sequence group B (infection after basic immunization, before booster with a total of three or four vaccinations; 6.3% of screened individuals, 15.6% of IMP) has the highest antibody concentration (median: 5706 BAU/mL, IQR: 3290–7257 BAU/mL) followed by group D (infection after booster; median 3846 BAU/mL, IQR 2536–6237 BAU/mL, 59.4% of screened individuals, 65.2% of IMP), group C (infection after basic immunization, a total of two vaccinations; median 3083 BAU/mL, IQR 1927–4599 BAU/mL, 12.5% of screened individuals, 8.0% of IMP) and group A (infection before 1st vaccination with ≥2 vaccinations; 2402 BAU/mL, 4.9% of screened individuals, 4.9% of IMP). Also, the proportion of individuals meeting the threshold of 4000 BAU/mL was higher in sequence group B (65%) compared with only 24% in group A, 31% in group C, and 48% in group D (*p* < 0.001; Chi‐square test). There were no differences between homologous and heterologous vaccination schemes (Figure 2F). As RNA vaccines were primarily used, we cannot conclude whether the type of vaccine has an influence on antibody titers.

Our approach focused on selection using QuantiVac. However, other high‐throughput assays can also be used, provided they quantitatively resolve the individuals with very high titers, e.g., with Elecsys tests, which showed a very good correlation with the QuantiVac ELISA results (Figure 2G). In contrast, other tests, namely the GenScript surrogate neutralization test against wildtype spike, are less suitable due to a ceiling effect. Here, the majority of donors had an inhibition above 95%, and those with particularly high titers could not be identified (Figure 2H). Not all donors with high neutralization capacity in the GenScript assay against Omicron also exhibit high anti‐SARS‐CoV‐2 concentrations. However, all donors with high anti‐SARS‐CoV‐2 antibody concentrations also neutralize very well in the GenScript assay (Figure 2I).

### 3.3 Characterization of collected CCP units and dynamics of anti‐SARS‐CoV‐2 antibodies over time in repeat plasmapheresis donors

The median number of donations per donor was 2 (IQR 1–3). Seventy‐six donors (44.2%) donated once, 40 donors (23.3%) twice, 17 donors (9.9%) donated thrice, and 39 donors donated 4–14 times. The median interval between the last immunization event and the eligibility check was 50 days (IQR 34.0–71.0) and from the eligibility check to the first donation was 21 days (IQR 12–38), between the first and second donation was 11 days (IQR 7.0–18.75), and the median interval between the first and last donation was 33.5 days (IQR 14.0–81.75). The median number of units collected per apheresis was 3 (IQR: 3–3). Among those who showed up for a subsequent donation, 40 of 238 subsequent donations had antibody levels below the threshold.

**FIGURE 3:**
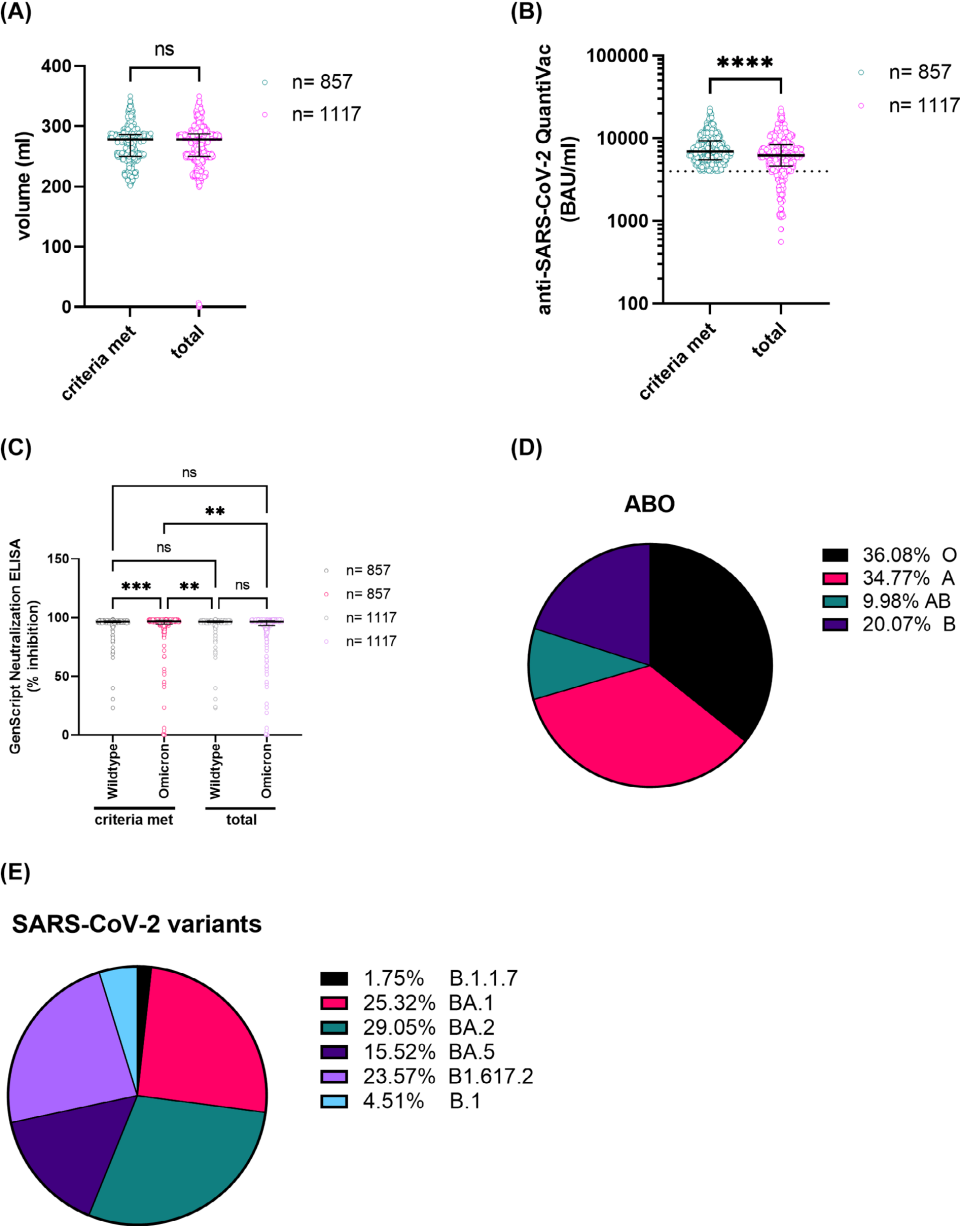
Characterization of collected CCP units. (A) Volume and (B) anti‐SARS‐CoV‐2 antibody concentrations of the compliant CCP and total collected CCPs. (C) Neutralization capacity of total and compliant CCP was assessed using GenScript Surrogate Neutralization Test against wildtype and Omicron. The horizontal solid lines indicate the median and the error bars the interquartile ranges. Statistical differences were assessed using Mann–Whitney (A‐B) or Kruskal‐Wallis test followed by Dunn's test for correction of multiple comparisons (C). p‐values <0.05 were considered statistically significant; ns = not significant, **p < 0.05; ***p < 0.005; ****p < 0.0005). (D) AB0 distribution of CCP meeting specifications and (E) the proportion of virus variants with which the donors were probably infected with. The variant that had caused the infection was assumed on the basis of the variant that was dominant in the population at the time of infection of the donors (population surveillance data of the Robert Koch Institute). [Color figure can be viewed at wileyonlinelibrary.com]

From January 2022 until May 2023, a total of 450 plasmapheresis sessions from 172 of the 224 eligible donors took place, resulting in 1117 CCP units with a median volume of 278 mL (Figure 3A) and a median antibody concentration of 6234 BAU/mL (Figure 3B). From these, 857 units met the specifications as IMP, with a median concentration of 6858 BAU/mL (Figure 3B). The main reason for not meeting the specification was the failure to reach the required antibody cut‐off (Figure 1). The ABO distribution of the IMP and the proportion of virus variants with which the donors were probably infected are shown in Figure 3D,E.

**FIGURE 4:**
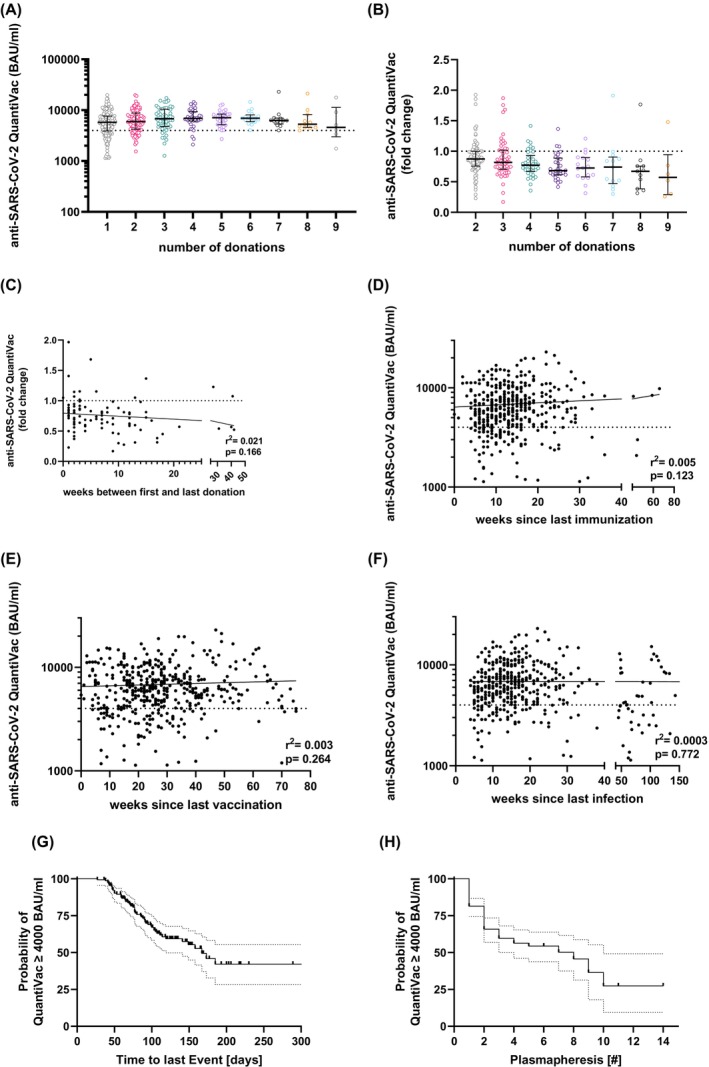
Anti‐SARS‐CoV‐2 antibody concentrations over time of donors who have donated at least once. Antibody titers at the respective donations are given as absolute values (A) or as ratio to the first donation (B). Dotted horizontal line indicates threshold of 4000 BAU/ml (A) or value of 1 (B). Values below 1 indicate a decline and values of 1 or higher indicate stable or increasing antibody titers. The horizontal solid lines indicate the median and the error bars the interquartile ranges (0 donations = eligibility check; gray triangles: 17 donors were wrongly invited as they were below the cut‐off at the preliminary examination). Statistical differences were assessed using Kruskal‐Wallis test followed by Dunn's test for correction of multiple comparisons and showed no statistical significance (*p* > 0.05). (C) Ratios of anti‐SARS‐CoV‐2 antibody concentrations of first and last donation of repeat donors irrespective of total number of donations. anti‐SARS‐CoV‐2 antibody concentrations in relation to (D) weeks since last immunization, (E) weeks since last vaccination, and (F) weeks since last infection. Solid line shows linear regression. The Kaplan–Meier estimate show the probability of having a titer of ≥4000 BAU/ml measured by QuantiVac depending on the interval between donation and last immunization event (G) or number of donations and (H) The Kaplan‐Meier analysis included donors whose anti‐SARS‐CoV‐2 antibody concentration initially exceeded the threshold of 4000 BAU/ml. T he first donation below 4000 BAU/ml was classified as an event. [Color figure can be viewed at wileyonlinelibrary.com]

Some of the donors donated several times. Some of the donors, who were still above the limit value at the eligibility examination or the first plasmapheresis, no longer had sufficient concentration of antibodies measured on occasion of a subsequent plasmapheresis (Figure 4A–C). Considering the interval between first and last donation (Figure 4C) or the interval between antibody determination and the last immunization (Figure 4D), regardless of whether the last immunization was a vaccination (Figure 4E) or an infection (Figure 4F), it was still possible to obtain CCPs above the cut‐off, but the number of eligible donors decreased. Kaplan–Meier estimates show that the probability of having a titer ≥4000 BAU/mL decreases the longer the interval since the last immunization event (Figure 4 G) and the number of donations (Figure 4H). The median time from the last immunization event and donation is 46 days (IQR 34–71).

### 3.4 Cross‐neutralization of CCP

The evolution of new virus variants raised concerns whether CCP units obtained might no longer be as potent in terms of neutralization against new variants as compared with the variant the donors had been infected with. The GenScript Neutralizing Assay (Figure 3C) demonstrated good neutralizing activity against B.1. (geo. mean: 93.97%) while neutralizing activity against Omicron was significantly lower (geo. mean: 68.23%).

**FIGURE 5:**
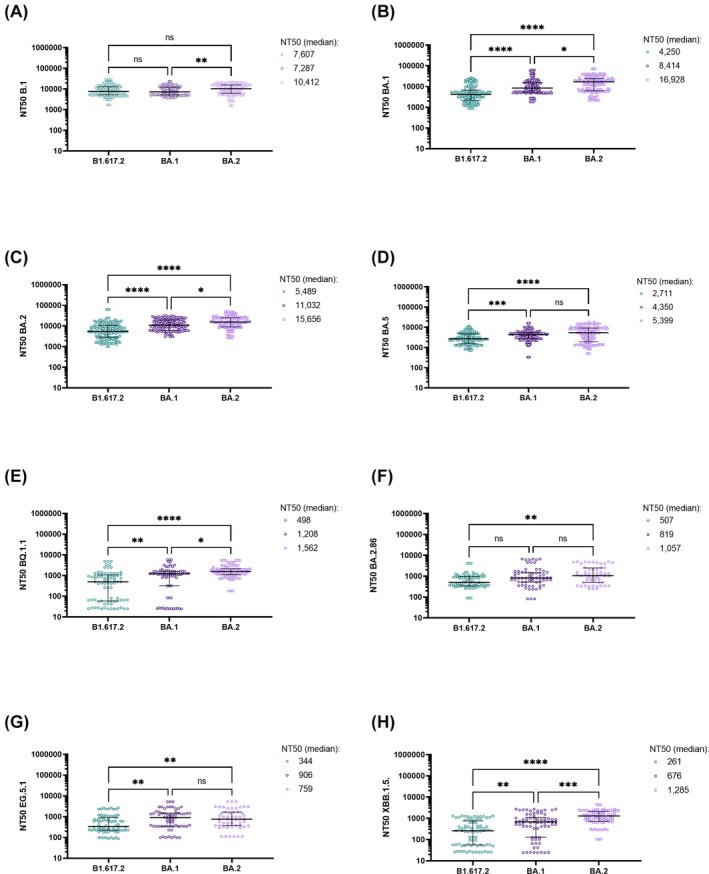
NT50 titers of CCPs meeting the specification whose donors were infected during the Delta (B1.617.2), BA.1 or BA.2 wave stratified by pseudovirus neutralization assay. The horizontal solid lines indicate the median and the error bars the interquartile ranges. Statistical analysis was performed using a Kruskal‐Wallis test and Dunn correction for multiple group comparisons. ns = not significant, *p < 0.05; **p < 0.05; ***p < 0.005; ****p < 0.0005). [Color figure can be viewed at wileyonlinelibrary.com]

The NT50 of CCPs stratified by donors that were infected during the B1.617.2, BA.1, or BA.2 wave were assessed by pseudovirus neutralization assay (Figure 5). These CCPs showed very good cross‐neutralization of B.1 and of the Omicron variants BA.1, BA.2, and BA.5, with CCPs from B1.617.2 donors being slightly less potent compared with the Omicron donors (Figure 5A–D). However, the neutralization capacities of the newer variants XBB.1.5, BQ.1.1, BA.2.86, and EG5.1 had fallen significantly (Figure 5E–H).

## 4 DISCUSSION

The COVIC‐19 study differs from most clinical CCP studies, in which the requirements for anti‐SARS‐CoV‐2 antibodies are lower, partly due to our strict criteria for donor/product selection^4^ to ensure treatment with high‐titer CCP in the study. As these criteria were applied for the first time in the COVIC‐19 study, empirical data on the feasibility of acquiring sufficient CCP units meeting such stringent criteria were not yet available. While many studies document CCP collection during early pandemic waves,^25^, ^26^ before vaccines were available, there is limited data on sourcing plasma from superimmunized donors, namely, those with very high antibody levels due to both infection and vaccination. Several studies suggested a dose effect of anti‐SARS‐CoV‐2 antibodies.^5^, ^27^, ^28^, ^29^, ^30^, ^31^, ^32^ One of the lessons learned from the pandemic is that when passively transferring antibodies with convalescent plasma, preference should be given to plasma units with very high antibody titers.^3^, ^33^ The evidence‐based dosage of CCP is of crucial importance^34^ —and thus the design of good clinical trial protocols starts with the feasibility of ambitious targets for CCP quality. COVIC‐19 is one of the few randomized trials which used CCP with pre‐specified very high antibody concentrations from superimmunized donors.^4^ The trial demonstrated a significant increase of anti‐SARS‐CoV‐2 antibody concentrations in CCP recipients and provided evidence that early administration of high‐titer CCP can prevent hospitalization or death in immunocompromised patients with mild COVID‐19.

The study shows that it is feasible to collect CCP for a randomized clinical trial with very high titers to allow adherence to a strict treatment protocol. However, it requires initial screening of a much larger donor pool, as a substantial proportion of potential donors are excluded due to antibody levels below the threshold (4000 BAU/mL; 58.6%) or anti‐leukocyte antibodies (16.1%). This is in line with other studies that also reported anti‐leukocyte antibodies as a major exclusion criterion.^35^ They have also reported anti‐leukocyte antibodies in donors without a history of alloimmunization, which has also led to the hypothesis that SARS‐CoV‐2 infection leads to more anti‐leukocyte antibodies.^36^ However, this is controversial.^37^ Also, waning of immunity, as observed in a subgroup of our donors, has been reported by others.^35^, ^38^ To avoid the collection of CCP units that cannot be used as an IMP, people who are willing to donate should undergo a stringent clearance investigation before the first plasmapheresis is performed. Despite these constraints, the project was supported by strong public willingness to donate.^39^ Overall, 224 individuals met all eligibility criteria; however, only 172 individuals donated CCP, namely, only one‐third of those who had presented for the first screening visit. This drop‐off was largely due to logistical issues such as illness, scheduling conflicts, or COVID‐19 exposure prior to donation.

Donor demographics do not provide a suitable selection criterion. Like others reported for non‐vaccinated CCP donors,^15^, ^40^, ^41^ we could not find a correlation between ABO blood group and antibody concentrations in our superimmunized donors either. Others have reported that older age, male sex, and hospitalization might be used as a proxy of high antibody titers.^42^ However, this was not a consistent finding^15^ and as shown by our CCP collections, might no longer apply for superimmunized donors.

By contrast, characteristics of the immunization history were more predictive: higher antibody concentrations and stronger cross‐neutralization were seen in individuals with multiple immunization events, particularly those who experienced infection after two vaccine doses and received booster vaccination afterwards. For example, 65% of individuals with two vaccinations, followed by infection and a booster, met the antibody threshold, compared with only 24% of those infected before their first vaccination. In time‐constrained or resource‐limited settings, focusing on donors with this immunization profile may improve efficiency. Other studies have already shown that vaccination and infection from either previous pre‐omicron variants, or omicron variants, lead to much higher neutralizing antibody titers.^43^ In our study, we additionally take into account the implications of the order in which immunization events occur.

A subgroup of donors met all criteria for repeat donations. Given that each plasmapheresis session provides two to three plasma units, a relatively small number of well‐selected repeat donors can meet supply needs. However, in line with other reports,^8^, ^44^ we also saw a drop in antibody concentration over time, and in 14.4% of follow‐up plasmaphereses, the antibody concentrations had fallen below the threshold in the interval. In our series, the highest antibody concentrations were observed in those with a breakthrough infection after vaccination, followed by a booster vaccination. Others have reported that SARS‐CoV‐2 breakthrough infection in vaccinated donors boosts nAb titers against VOCs Delta and Omicron, but such titers decay shortly after infection.^45^ Therefore, plasmapheresis should occur soon after screening to optimize yield. Given the observed rapid decline in high‐titer donors, targeted booster vaccinations may be a viable strategy to re‐evaluate antibody levels in motivated individuals should a shortage of eligible donors occur. Studies have shown that additional vaccine doses can significantly enhance binding and neutralizing antibody titers, including cross‐reactive responses.^7^, ^12^, ^13^ However, any recommendation should be balanced against national guidelines, donor safety, and ethical considerations. A personalized approach, namely, offering boosters to previously high‐titer donors, could help sustain the donor pool but should only be done within a controlled immunization program. With a rapidly changing virus such as SARS‐CoV‐2, recruiting new donors with current infections may also be important: on the one hand, to keep the interval to the immunization event (infection) short, but above all to reflect immunity to new variants in the donor pool, and thus maintain a contemporary CCP pool. Our recruiting strategy via social media and internet calls enables criteria to be adjusted promptly by specifically calling on donors who have recently received booster vaccinations or had infections to come and register.

Our data also demonstrate that collection of new CCP units from donors infected with more recent SARS‐CoV‐2 variants provides cross‐neutralization of new VOCs. This supports continued CCP collection during ongoing outbreaks to ensure variant coverage in plasma stocks.^46^, ^47^ In clinical practice, stratification of CCP units according to the suspected variant is not feasible due to logistical constraints and lack of standardized, variant‐specific assays. Furthermore, our data show that high‐titer CCP has a broader neutralization capacity.^7^ Until standardized, high‐throughput variant‐specific assays are available, selecting CCP on high antibody thresholds and confirmed infection and vaccination history remains the most practical approach. Ideally, donation should occur shortly after the latest immunization event to ensure optimal antibody levels.

Biochemical assays identifying high anti‐SARS‐CoV‐2 antibody concentrations reliably correlate with neutralizing titers against multiple variants, consistent with previous reports.^7^, ^8^, ^42^, ^48^ Once this correlation is established, high‐throughput assays can serve as effective screening tools, reducing reliance on labor‐intensive neutralization tests. The use of standardized high‐throughput biochemical tests can contribute to quality assessment and harmonization of laboratories and clinical trial groups worldwide.^48^ Given regional regulatory differences and recommendations, it is important to contextualize the antibody thresholds employed in our study. While the U.S. FDA^49^ recommends the Ortho VITROS IgG quantitative assay with >200 BAU/mL as the high‐titer cut‐off under FDA guidance, the Roche Elecsys Anti‐SARS‐CoV‐2 S assay defines high antibody levels at >210 U/mL. Both assays are WHO‐calibrated and report in standardizable units, allowing direct comparison. In contrast, GenScript's cPass surrogate neutralization assay uses functional inhibition (≥80%) as a criterion, without providing quantifiable antibody concentrations. The >4000 BAU/mL cut‐off based on the QuantiVac assay employed in our study strongly exceeds the thresholds recommended by the FDA^49^ and is based on prior data correlating high BAU/mL levels with potent neutralizing capacity.^7^ These findings support ongoing discussions suggesting that the FDA's currently recommended antibody thresholds may underestimate functional potency and warrant reassessment.^34^, ^50^ The use of WHO‐standardized quantitative assays and elevated selection criteria may help improve the reliability and efficacy of CCP products.

To summarize, the collection of very high‐titer anti‐SARS‐CoV‐2 immune plasma from superimmunized donors is feasible but requires substantial donor selection, rapid screening, and prompt apheresis to capitalize on the short period of very high titers. The in vitro analyses demonstrate that these CCP units contain very high concentrations compared with CCP used in previous trials and have broad variant neutralization. This is in line with other reports that anti‐spike immunoglobulin levels predict high levels of neutralizing antibodies and can be used as a criterion for procurement of high‐titer CCP^35^, ^51^, ^52^—including new variants by which the donors have not been infected. This strategy can inform future pandemic preparedness and optimize convalescent plasma programs by enabling early collection of high‐titer plasma for immunotherapy‐sensitive pathogens.

## CONFLICT OF INTEREST STATEMENT

The Authors declare no conflict of interest.
